# The knobby ball illusion

**DOI:** 10.1177/20416695231165182

**Published:** 2023-03-22

**Authors:** Peter U. Tse, Vincent Hayward

**Affiliations:** Psychological and Brain Sciences, 3728 Dartmouth College, Hanover, NH, USA; Actronika SAS, Paris, France; Institut des Systèmes Intelligents et de Robotique, Sorbonne Université, Paris, France

**Keywords:** haptics/touch, 3D perception, contours/surfaces, shape

## Abstract

A novel haptic illusion is described where deformations of the fingertip skin lead to subsequent misperceptions of an object's shape.

Here we describe an easy way to elicit a haptic illusion called the “knobby ball” or “knobby sphere” illusion that works well in the classroom. The observer deforms his or her skin from the indentation caused by an object with edges, then feels a small ball and notices that it no longer feels round, but instead seems rather knobby, as if it had an irregular shape or had corners as if it were a polyhedron.

The observer should take a pencil that has a hexagonal cross-section and press it as firmly as possible between his or her index finger and thumb for about a minute. In the other hand they should hold a small hard sphere, such as a large ball bearing or small marble of approximately 3 mm diameter so that they can quickly hand it over to the right hand. After a deep angular indentation in the skin has formed, it is important to immediately pass the small ball to the finger with the deformed skin. When the subject rolls the ball back and forth, into and out of the groove left by the pencil between his or her finger and thumb for a few seconds, the ball feels knobby. The best way to experience this illusion is to have two balls, one of which stays in the original hand between the thumb and index finger, and the other which goes to the same location in the hand that held the pencil. The perceived difference between these two balls is quite marked, one feeling like a sphere, the other knobby. Figure 1 shows an example of household objects that can be used to elicit the illusion.

**Figure 1.**
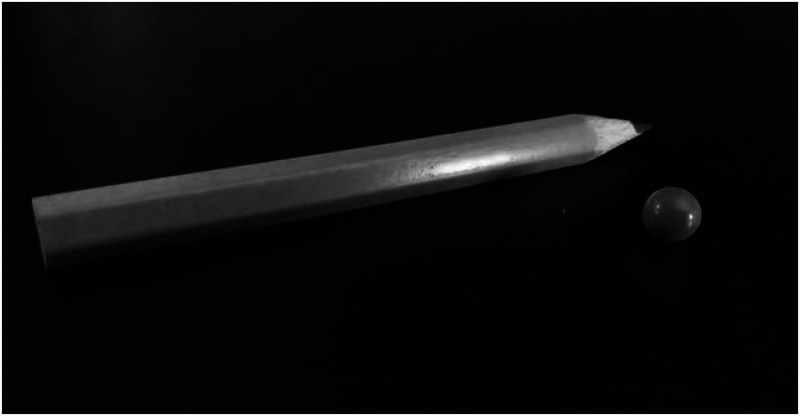
Examples of objects able to elicit the illusion.

In an informal two-alternative forced choice psychophysical procedure, 14 subjects, when asked “Which side feels rounder?” in the two ball version of this task, reported that the ball in the original hand felt rounder than the ball held in the hand with the pencil-induced indentation. In all cases, observers knew that they were in fact holding two small balls, but all reported feeling that the ball in the hand with the deformed haptic sheet no longer felt fully spherical. For half of these observers the pencil was held in the right hand, and for the other half the pencil was held in the left hand.

Why does this illusion occur? Three hypotheses present themselves as reasonable: (1) The viscoelastic properties of the fingertip tissues are weakly taken into account by the somatosensory system; (2) Another possibility is that slowly adapting mechanoreceptors in the skin eventually adapt more in the regions of high curvature, leading to the misperception of shape once the edged stimulus is withdrawn and replaced by a smooth one; (3) The effect could conceivably be a haptic after-effect, perhaps due to adaptation at the level of haptic curvature detectors along the somatosensory neural pathway.

There are previous examples of tactile after-effects. The most relevant was reported by Vogels et al. (1997). There participants touched curved objects and experienced a negative curvature after-effect. These authors wrote several papers investigating this effect, summarized in the review by Hayward (2008). An explanation in terms of (3) based on cortical or subcortical haptic after-effects is unlikely, however, because the reported after-effect is positive. That is, the sensation persists, unlike with other after-effects known in vision or in audition (or that of Vogels et al.), which are characterized by sensation “reversals” following adaptation.

An explanation of type (2) is also unlikely because the time-scale of mechanoreceptor adaptation is much too brief; generally less than 1 s, even for the so-called slowly adapting units (Johansson & Vallbo, 1983). Units with longer time scales are found only in the hairy skin (Vallbo et al., 1999), and here the fingertips were used.

This leaves an explanation of type (1) as the most likely candidate. Fingertip skin biomechanics exhibits relaxation time scales that can be very long, on the order of 5–10 s (Wang & Hayward, 2007). This fact can be visually observed when pressing the finger on a sharp edge and then looking at the persisting imprint, which could explain the positive character of the effect at hand.

It is interesting that the illusory edges are not felt when the fingertip is in free air but is only felt when touching a smooth surface. Because touch signals must be present to infer the presence of a surface and its shape, it stands to reason that no shape or surface should be perceptually constructed in the absence of haptic input.

It is likely that the present effect is an example of a more general class of effects. If, for example, one creates a skin indentation with any sharp edge, say by pressing the edge of a box with a finger, and then touches a flat surface, the sensation of an edge survives for a few seconds.

If the somatosensory system is reconstructing a surface in light of incorrect assumptions, there should be other such illusions to be discovered. For example, tracing a circle or line of a certain objective size or length over the skin should lead to a given perception of size or length, while tracing the same circle or line over skin that has been stretched should lead to an altered perception of size or length.

Future work should explore deformations of sensory sheets, haptic and otherwise, as a probe to uncover the constructive processes and assumptions that allow cortical and subcortical processes to infer the structure of objects and events in the world and in our bodies.

## References

[bibr1-20416695231165182] Hayward V. (2008). A brief taxonomy of tactile illusions and demonstrations that can be done in a hardware store. Brain Research Bulletin, 75(6), 742–752. 10.1016/j.brainresbull.2008.01.008 18394520

[bibr2-20416695231165182] Johansson R. S. Vallbo A. B. (1983). Tactile sensory coding in the glabrous skin of the human hand. Trends in Neurosciences, 6, 27–32. 10.1016/0166-2236(83)90011-5

[bibr3-20416695231165182] Vallbo A. B. Olausson H. Wessberg J. (1999). Unmyelinated afferents constitute a second system coding tactile stimuli of the human hairy skin. Journal of Neurophysiology, 81(6), 2753–2763. 10.1152/jn.1999.81.6.2753 10368395

[bibr4-20416695231165182] Vogels I. M. Kappers A. M. Koenderink J. J. (1997). Investigation into the origin of the haptic aftereffect of curved surfaces. Perception, 26(1), 101–117. 10.1068/p260101 9196695

[bibr5-20416695231165182] Wang Q. Hayward V. (2007). In vivo biomechanics of the fingerpad skin under local tangential traction. Journal of Biomechanics, 40(4), 851–860. 10.1016/j.jbiomech.2006.03.004 16682045

